# Metabolomic alterations in human cancer cells by vitamin C-induced oxidative stress

**DOI:** 10.1038/srep13896

**Published:** 2015-09-09

**Authors:** Megumi Uetaki, Sho Tabata, Fumie Nakasuka, Tomoyoshi Soga, Masaru Tomita

**Affiliations:** 1 Institute for Advanced Biosciences, Keio University, 246-2 Mizukami, Kakuganji, Tsuruoka, Yamagata 997-0052, Japan; 2 Systems Biology Program, Graduate School of Media and Governance, Keio University, 5322 Endo, Fujisawa, Kanagawa 252-0882, Japan; 3 Environment and Information Studies, Keio University, 5322 Endo, Fujisawa, Kanagawa 252-0882, Japan

## Abstract

Intravenous administration of high-dose vitamin C has recently attracted attention as a cancer therapy. High-dose vitamin C induces pro-oxidant effects and selectively kills cancer cells. However, the anticancer mechanisms of vitamin C are not fully understood. Here, we analyzed metabolic changes induced by vitamin C in MCF7 human breast adenocarcinoma and HT29 human colon cancer cells using capillary electrophoresis time-of-flight mass spectrometry (CE-TOFMS). The metabolomic profiles of both cell lines were dramatically altered after exposure to cytotoxic concentrations of vitamin C. Levels of upstream metabolites in the glycolysis pathway and tricarboxylic acid (TCA) cycle were increased in both cell lines following treatment with vitamin C, while adenosine triphosphate (ATP) levels and adenylate energy charges were decreased concentration-dependently. Treatment with *N*-acetyl cysteine (NAC) and reduced glutathione (GSH) significantly inhibited vitamin C-induced cytotoxicity in MCF7 cells. NAC also suppressed vitamin C-dependent metabolic changes, and NAD treatment prevented vitamin C-induced cell death. Collectively, our data suggests that vitamin C inhibited energy metabolism through NAD depletion, thereby inducing cancer cell death.

High-dose vitamin C treatment has a controversial history as a potential chemotherapeutic agent for cancer treatment^1^. A number of previous reports have suggested that high-dose vitamin C has anticancer effects^2^,^3^ while in other studies, it has shown no benefits in patients with cancer^4^,^5^. In view of these controversies, vitamin C treatment has recently been re-evaluated as a potential cancer therapy^6^,^7^,^8^,^9^,^10^. These analyses have revealed that high-dose vitamin C is more cytotoxic to cancer than it is to normal cells^11^. Moreover, vitamin C induces death of various types of cancer cells including mesothelioma, pancreatic, and leukemia cells^10^,^12^,^13^. High-dose vitamin C suppressed tumor growth in animal models and tissue culture studies^10^,^12^ and, therefore, may indeed have applications as a novel treatment for various cancers.

High-dose vitamin C kills cancer cells by acting as a pro-drug, which delivers hydrogen peroxide (H_2_O_2_)^8^,^10^. Increased levels of reactive oxygen species (ROS) including H_2_O_2_ are thought to play an important role in the initiation and progression of cancer. Excessive levels of ROS are known to cause cellular damage including senescence via activation of protein kinase Cδ (PKCδ)^11^,^14^ and the release of cytochrome c from the mitochondria, leading to apoptosis^15^,^16^. Moreover, cellular ROS levels affect the redox status and metabolism^17^. H_2_O_2_ can change the ratio of oxidized glutathione (GSSG) and reduced (GSH) glutathione to a more oxidized state since H_2_O_2_ is reduced to water (H_2_O) by glutathione peroxidase (GPx)^18^. Previous reports also showed that vitamin C treatment induced cytotoxicity by adenosine triphosphate (ATP) depletion in some cancer cells^6^,^12^,^19^. Therefore, vitamin C-induced H_2_O_2_ may alter intracellular metabolism in cancer cells by disrupting the redox balance. However, the effects of vitamin C on metabolism, including glycolysis, the tricarboxylic acid (TCA) cycle, and the pentose phosphate pathway (PPP), have not been clarified. Furthermore, the biological significance of vitamin C-induced metabolic alterations is still unknown.

Therefore, in this study, we sought to determine the effects of vitamin C on cancer cell metabolism using capillary electrophoresis time-of-flight mass spectrometry (CE-TOFMS).

## Results

### High-dose vitamin C-induced cytotoxicity in cancer cells

**Figure 1:**
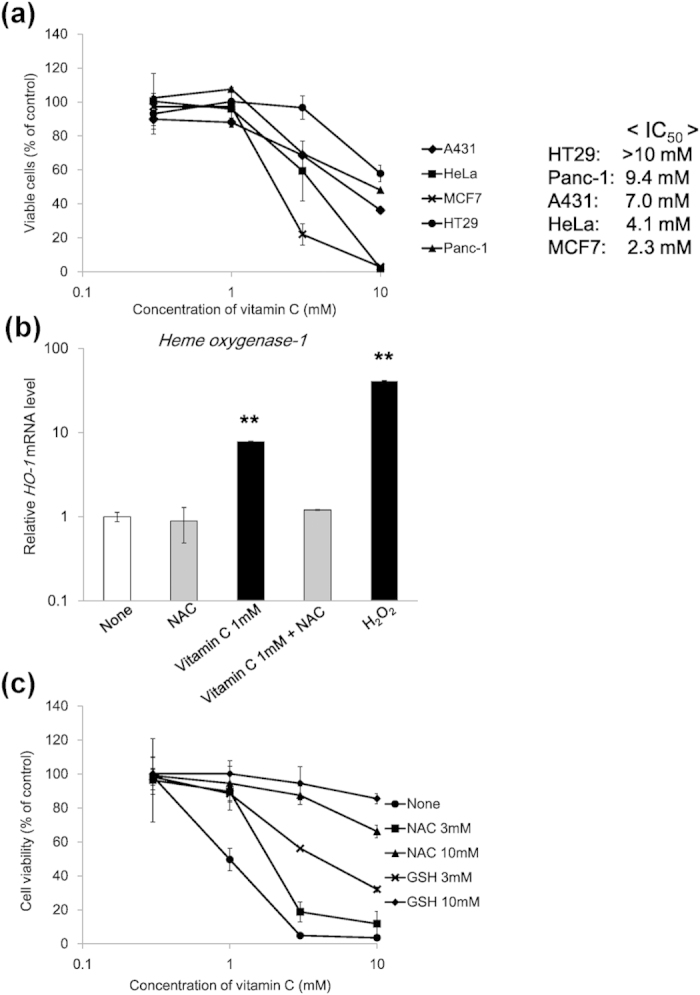
Effects of vitamin C-induced hydrogen peroxide (H_2_O_2_) on viability of cancer cells. (**a**) Cancer cells were treated with vitamin C for 2 h, washed, and cultured for an additional 46 h in DMEM in the absence of vitamin C. Cell viability was determined using MTT assays. IC_50_ values indicate the concentration of vitamin C that inhibited survival by 50%, as determined by MTT assays. (**b**) Effects of vitamin C on *HO-1* expression in MCF7 cells. Cells were treated with vitamin C (1 mM), NAC (10 mM), and H_2_O_2_ (1 mM) for 24 h. Expression levels of *HO-1* mRNA were measured using qPCR. (**c**) Suppressive effects of antioxidants NAC and GSH on vitamin C-induced cytotoxicity in MCF7 cells. Cell viability was determined using MTT assays in MCF7 cells treated without or with vitamin C and antioxidants. Data are presented as means ± SDs from triplicate experiments, ^**^P < 0.01.

High-dose vitamin C has been reported to show significant anticancer effects *in vitro* and *in vivo*^6^,^8^. To confirm the effects of vitamin C on the survival of A431, Panc-1, HeLa, HT29, and MCF7 cells, we examined cell viability using 3-(4,5-dimethylthiazol-2-yl)-2,5-diphenyltetrazolium bromide (MTT) assays. The results showed that cell viability was decreased following exposure to high concentrations of vitamin C (3 or 10 mM) in all cancer cell lines (Fig. 1a). The HT29 cells were the least sensitive to vitamin C with a half-maximal inhibitory concentration (IC_50_) of 10 mM or more, followed by Panc-1, A-431, HeLa, and MCF7 cells (IC_50_, 2.4 mM). These data suggests that high-dose vitamin C induced cytotoxic effects in cancer cells, albeit with varying efficacies.

Previous studies have reported that high-dose vitamin C induces H_2_O_2_^6^,^7^,^8^,^12^. Therefore, we assessed the oxidative stress response in MCF7 cells treated with vitamin C by examining the expression of hemeoxygenase-1 (HO-1), a cellular oxidative stress marker, using quantitative real-time polymerase chain reaction (qPCR). *HO-1* mRNA level in MCF7 cells significantly increased by vitamin C and H_2_O_2_, and this effect was suppressed by treatment with the antioxidant N-acetylcysteine (NAC, Fig. 1b). Furthermore, we investigated whether vitamin C induced cell death by generating H_2_O_2_ in MCF7 and HT29 cells. The antioxidants NAC and GSH attenuated the vitamin C-induced cytotoxicity in these cells (Fig. 1c), indicating that vitamin C-induced oxidative stress led to cancer cell death.

### Metabolomic profiles of MCF7 cells treated with vitamin C

**Figure 2:**
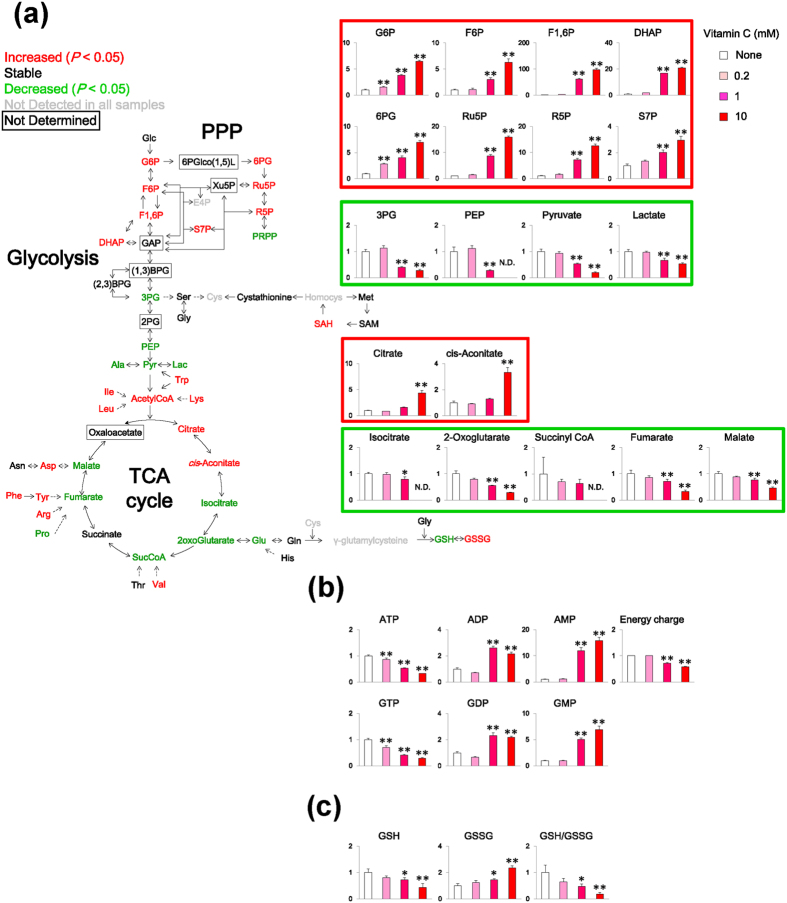
Vitamin C-induced metabolic alterations in MCF7 cells. (**a**) Metabolic alterations in glycolysis and the TCA cycle induced by vitamin C. MCF7 cells were incubated in DMEM without or with vitamin C, and metabolites levels were measured using CE-TOSMS. Colors of metabolites on heatmap indicate significant differences (red, upregulated; green, downregulated). Bar graphs indicate fold changes relative to control sample (None). (**b**) Effects of vitamin C on levels of AMP, ADP, ATP, GMP, GDP, GTP, and adenylate energy charge. Bar graphs indicate fold changes relative to control sample (None). Adenylate energy charge calculation: (ATP + 0.5 × ADP)/(ATP + ADP + AMP). (**c**) Effects of vitamin C on levels of GSH and GSSG and GSH:GSSG ratio. Bar graphs show relative metabolite levels compared to control (None). Data are presented as means ± SD of triplicate experiments, ^*^P < 0.05, ^**^P < 0.01. ND, not detected.

Next, we explored the effects of vitamin C on the metabolomic profile of MCF7 cancer cells using CE-TOFMS. The results revealed that following exposure to cytotoxic concentrations of vitamin C (≥1 mM) the levels of various metabolites were obviously altered in the MCF7 cells. Our analysis specifically revealed that the levels of the metabolites associated with the energy metabolism pathways examined, including those upstream of glycolysis, pentose phosphate pathway (PPP), and partial TCA cycle (citrate and cis-aconitate), were increased by the high-dose vitamin C (Fig. 2a). Conversely, the levels of metabolites downstream of glycolysis and the TCA cycle with the exception of citrate and cis-aconitate were decreased. ATP concentrations and adenylate energy charges were also decreased in a coordinated manner (Fig. 2b). These findings suggest that the high-dose vitamin C blocked the energy flux in glycolysis and the TCA cycle and consequently inhibited ATP production. Next, we examined whether the vitamin C-induced oxidative stress influenced the GSH redox balance. The levels of GSSG and GSH in the MCF7 cells were increased and decreased, respectively by vitamin C. In addition, the GSH/GSSG ratio was decreased at cytotoxic vitamin C concentrations and this effect was likely mediated by the associated generation of H_2_O_2_ generation, which may have affected the redox status of GSH (Fig. 2c). Furthermore, levels of amino acids, including Phe, Leu, Val, Ile, Lys, Trp, Ala, Tyr, Asp, and Arg, in MCF7 cells were increased following vitamin C treatment (Supplementary Figure 1A). The effects of cytotoxic concentrations of vitamin C on the metabolomic profiles of the HT29 cells were similar (MCF7 cells ≥1 mM, HT29 cells 10 mM, Supplementary Figure 2A–D).

### Effects of NAC on vitamin C-dependent reduction in energy metabolism in MCF7 cells

**Figure 3:**
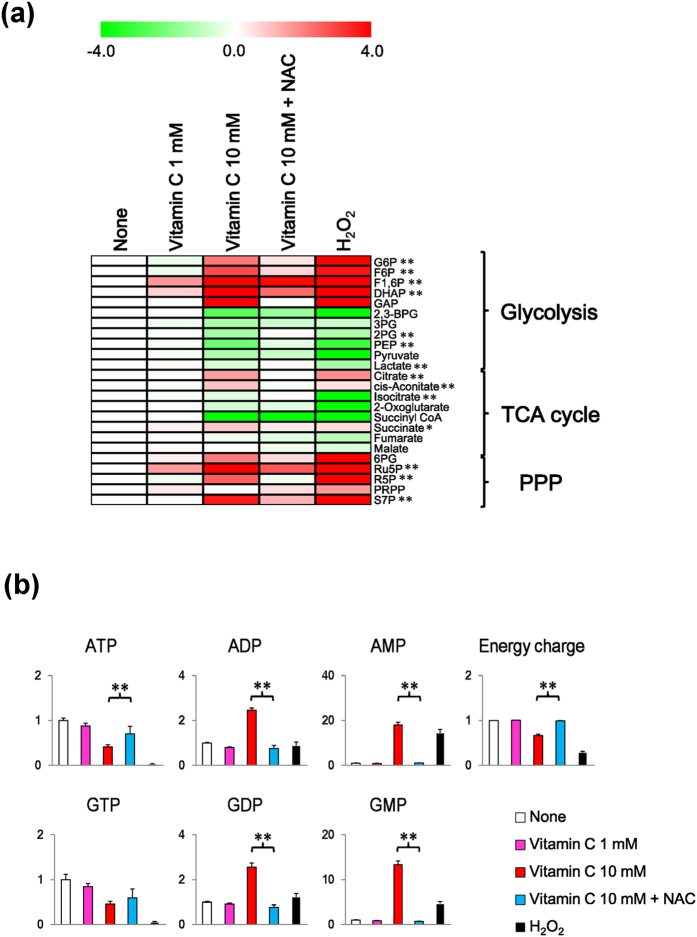
Effects of N-acetyl cysteine (NAC) on energy metabolism in MCF7 cells treated with vitamin C. (**a**) Effects of NAC on metabolites of glycolysis, the TCA cycle, and the PPP in MCF7 cells stimulated with vitamin C. Heatmap depicts log2-transformed ratios of measured sample to control sample (None) concentrations. ^*^P < 0.05, ^**^P < 0.01 (comparing lanes 3 and 4). (**b**) Effects of NAC on levels of AMP, ADP, ATP, GMP, GDP, GTP, and adenylate energy charge. Bar graphs indicate fold changes relative to control sample (None). Adenylate energy charge calculation: (ATP + 0.5 × ADP)/(ATP + ADP + AMP). Data are presented as means ± SD of triplicate experiments. ^**^P < 0.01.

To examine whether the high-dose vitamin C-induced H_2_O_2_ inhibited energy metabolism, we analyzed the metabolomic profiles of MCF7 cells treated with vitamin C and the antioxidant NAC. The results revealed that most of the vitamin C-induced metabolic changes in glycolysis, the TCA cycle, and the PPP were abolished by NAC treatment (Fig. 3a). In addition, ATP concentrations and adenylate energy charges were restored more by cotreatment with NAC than with vitamin C treatment alone (Fig. 3b). Interestingly, the metabolite profiles observed following vitamin C treatment were similar to those following H_2_O_2_ treatment (Fig. 3a). The changes in amino acid levels induced by vitamin C were also suppressed by NAC (Supplementary Figure 1B). These results suggest that vitamin C modulated energy metabolism by generating H_2_O_2_.

### Vitamin C-induced H_2_O_2_ depleted nicotinamide adenine dinucleotide (NAD) in MCF7 cells

**Figure 4:**
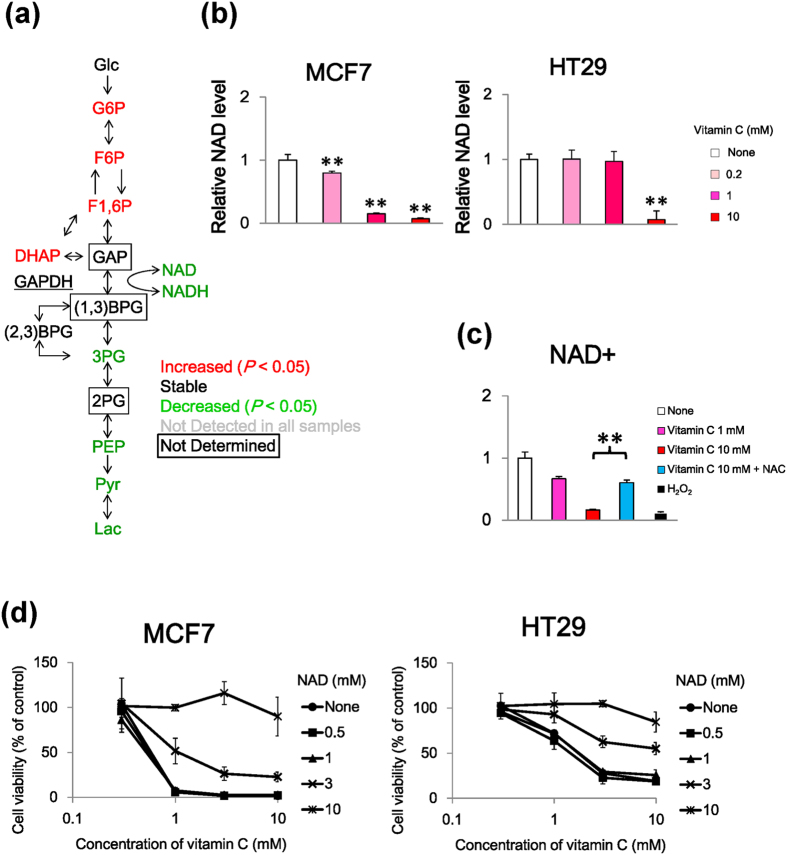
Nicotinamide adenine dinucleotide (NAD) depletion induced by vitamin C-induced H_2_O_2_ in MCF7 cells. (**a**) Metabolite map of glycolysis. Colors of metabolites indicate significant differences (red, upregulated; green, downregulated). Conversion of GAP to 1,3- BPG mediated by GAPDH. (**b**) NAD levels were decreased by vitamin C in MCF7 (left) and HT29 cells (right) and were determined using CE-TOFMS. Bar graphs indicate fold changes relative to control sample (None). (**c**) Effects of NAC on levels of NAD in MCF7 cells. Bar graphs show metabolite levels relative to those of control (None). (**d**) Effects of NAD on viability of MCF7 (left) and HT29 (right) cells determined by MTT assay in both cell lines treated without or with vitamin C and NAD. Data are presented as means ± SD of triplicate experiments, ^**^P < 0.01.

We found that vitamin C caused metabolic alterations in glycolysis and depleted ATP in MCF7 and HT29 cells (Fig. 2a,b, Supplementary Figure 2A and 2B). Intriguingly, the levels of the metabolites upstream of glycolysis in the MCF7 and HT29 cells were augmented following treatment with vitamin C while those downstream were reduced (Fig. 2a and Supplementary Figure 2A). Analysis of the metabolic profiles of the components of glycolysis suggested that the glycolytic flux between glyceraldehyde 3-phosphate (GAP) and D-glycerate 1,3-bisphosphate (1,3-BPG) mediated by glyceraldehyde 3-phosphate dehydrogenase (GAPDH) may have been suppressed by vitamin C in the MCF7 and HT29 cells (Fig. 4a). To investigate whether metabolic changes induced by vitamin C were related to GAPDH, its expression was assessed using qPCR and was revealed to be unaffected by vitamin C or H_2_O_2_ treatment in MCF7 cells (Supplementary Figure 3). The treatment of U937 cells, which are derived from a human histiocytic lymphoma cell line, with H_2_O_2_, inactivates GAPDH via nicotinamide adenine dinucleotide (NAD) depletion^17^. Therefore, we examined the intracellular NAD levels in vitamin C-treated MCF7 cells treated, and discovered the levels were decreased, and this effect was reversed by NAC (Fig. 4b,c). These data suggest that high-dose vitamin C may inhibit glycolysis through NAD depletion. Moreover, we investigated whether vitamin C caused cell death through NAD depletion in MCF7 and HT29 cells. Our data showed that NAD suppressed the vitamin C-induced cell death in both cell lines (Fig. 4d). Taken together, these data suggest that vitamin C-induced oxidative stress inhibited the glycolytic flux by NAD depletion and consequently caused cell death.

## Discussion

In this study, we examined the effects of vitamin C on the metabolomic profiles of different cancer cells. Our data showed that high-dose vitamin C was cytotoxic in the cancer cell lines investigated and altered the levels of various metabolites. Therefore, these results suggest that vitamin C may indeed have applications as a potential anticancer therapeutic agent.

Numerous laboratories have reported that high-dose vitamin C treatment induces cell death by H_2_O_2_ generation^6^,^10^,^12^,^16^,^20^. In addition, H_2_O_2_ is involved in the maintenance of the redox status including the GSH/GSSG ratio and, is, therefore, expected to affect metabolism. In this study, we showed that levels of the upstream metabolites of glycolysis and TCA cycle were increased by vitamin C, while levels of those downstream were decreased. Additionally, ATP levels were decreased by vitamin C in the cancer cells tested, suggesting that vitamin C inhibited energy metabolism. We also found that cotreatment with NAC reversed the inhibitory effects of vitamin C on glycolysis, the TCA cycle, and the PPP; these results confirmed that vitamin C disrupted energy metabolism by H_2_O_2_ generation. Finally, we found that NAD depletion was critical for the observed effects on glycolytic metabolism and subsequent induction of cell death. Therefore, our results showed that vitamin C-induced oxidative stress inhibited energy metabolism through NAD depletion and consequently caused cytotoxicity.

In this study, we found that the different cancer cell lines showed varying sensitivities to vitamin C and considered that this phenomenon may be regulated by multiple factors, such as redox system, transporter expression, and hypoxia condition. For instance, we examined the cancer cell line death following treatment with H_2_O_2_ (data not shown) and discovered that its cytotoxicity was not correlated with that of vitamin C in these cell lines. Chen *et al.*^6^ reported no correlations between vitamin C-induced cell death and GSH, catalase, or GPx activities. Meanwhile, the vitamin C transporter was involved in the sensitivity of breast cancer cells to vitamin C^21^,^22^. Furthermore, hypoxic conditions (1% O_2_) suppressed the cytotoxicity of vitamin C more in 60 cancer cell lines than normoxic conditions (21% O_2_) did^23^.

In this study, we report the first demonstration of vitamin C-induced changes in metabolomic profiles. Recent studies have shown that cancer cell metabolism may be a possible target for therapy. Cancer cells reprogram their metabolic processes according to the tumor microenvironment or cancer progression^24^,^25^,^26^. Previous studies have also reported that oncogenic signals such as Ras and c-Myc activity regulate the expression of metabolic enzymes, and thereby contribute to tumor development^25^,^27^,^28^. Moreover, cancer metabolism is characterized by abnormal energy production, known as the Warburg effect^29^,^30^,^31^,^32^,^33^. ATP generation in cancer cells shifts from oxidative phosphorylation to glycolysis, even under normoxic conditions. Therefore, glycolysis in cancer cells may be a potential target for cancer therapeutics. In our study, we found that vitamin C inhibited glycolysis by depleting NAD. Additionally, several reports have indicated that vitamin C therapy selectively kills cancer cells^8^,^21^. Therefore, since ATP production in cancer cells is more strongly dependent on glycolysis than it is in normal cells, the effects of vitamin C on survival may be more dramatic in cancer cells.

We found that vitamin C distinctly altered the pattern of the glycolytic metabolites. The GAPDH-mediated reaction between GAP and 1,3-BPG revealed that the upstream glycolytic metabolites were increased while those downstream decreased, in response to vitamin C treatment. GAPDH expression reportedly increased in several tumor types including prostate, breast, and lung cancers^34^,^35^,^36^. Furthermore, GAPDH expression is upregulated via activation of the hypoxia-inducible factor (HIF-1) in breast cancer cells^37^. However, GAPDH expression in MCF7 cells was not increased following vitamin C treatment. Since the vitamin C-induced metabolic changes occurred within 1 h following treatment, the changes in the expression of metabolic enzymes may not be involved. On the other hand, vitamin C suppressed the NAD levels by generating H_2_O_2_ in MCF7 and HT29 cells, and vitamin C-induced cell death was reversed by NAD supplementation in both cell lines. These data suggest that NAD depletion may trigger vitamin C-induced cell death in cancer cells. In addition, Chen *et al.*^6^ hypothesized that vitamin C-induced H_2_O_2_ causes DNA damage, leading to enhanced PARP activation, which may consume NAD and deplete ATP^7^. Our metabolomics studies supported this mechanism.

In conclusion, our findings suggest that vitamin C promoted cancer cell death by inhibiting energy metabolism via NAD depletion, induced by H_2_O_2_ generation. Further investigations are required to elucidate the specific mechanisms by which NAD depletion mediates the vitamin C-induced cytotoxicity. In addition, studies to confirm whether this pathway may be a potential target and thereby contribute to the improvement of cancer therapy would be expedient.

## Methods

### Regents and cell culture

Vitamin C, NAC, GSH, and NAD were purchased from Sigma-Aldrich (St. Louis, MO, USA). Human skin epidermoid carcinoma (A-431), human breast mammary gland adenocarcinoma (MCF7), and human colon colorectal adenocarcinoma (HT29) cells were obtained from the American Type Culture Collection (ATCC, Manassas, VA, USA). Human cervix carcinoma (HeLa) cells were obtained from the Japanese Collection of Research Bioresources (Tokyo, Japan), while human pancreas adenocarcinoma (Panc-1) cells were purchased from the RIKEN BioResource Center (Tsukuba, Japan). All the cells were grown in Dulbecco’s modified Eagle’s medium (DMEM, Nissui Pharmaceuticals Co., Ltd., Tokyo, Japan) supplemented with 10% fetal bovine serum, 100 U/mL penicillin, 100 mg/mL streptomycin, and 0.25 mg/mL amphotericin B at 37°C in a humidified atmosphere with 5% CO_2_.

### Cell viability assay

The cell viability was measured using the MTT assay as follows. The cells (7.5 × 10^3^) were seeded in each well of a 96-well plate and incubated for 24 h. Then, vitamin C was added and the cells were further incubated for 2 h, washed, and then cultured for an additional 46 h in DMEM in the absence of vitamin C. The cells were pretreated with NAC, GSH, and NAD 1 h prior to the incubation with vitamin C. Then, 50 μL of the MTT reagent (2 mg/mL in phosphate-buffered saline, PBS) was added to each well, and the plates were incubated for an additional 2 h. The resulting formazan crystals were dissolved in 100 μL of dimethyl sulfoxide (DMSO) after the culture medium had been aspirate nm using a TECAN microplate reader with Magellan software (Männedorf, Switzerland).

### qPCR analysis

RNA was extracted from vitamin C-treated MCF7 cells using TRIzol (Life Technologies, Gaithersburg, MD, USA) according to the manufacturer’s protocol, and 1 μg was reverse transcribed using a first-strand cDNA synthesis kit (ReverTra Ace α, Toyobo Co., Ltd., Osaka, Japan). The qPCR was performed using the SYBR premix Ex Taq (Takara, Shiga, Japan) on a StepOne Plus Real-Time PCR system (Applied Biosystems, Foster City, CA, USA) according to the manufacturer’s instructions. Quantification was performed using the ΔΔCt method, and *RPL27* expression used as an internal reference. The melt curve analysis confirmed that all the qPCR products were generated in the form of double-stranded DNA. The primers used were as follows: HO-1, 5′-CGGGCCAGCAACAAAGTGCAAG-3′ (sense) and 5′-GTGTAAGGACCCATCGGAGAAG-3′ (antisense) and RPL27, 5′-CTGTCGTCAATAAGGATGTCT-3′ (sense) and 5′-CTTGTTCTTGCCTGTCTTGT-3′ (antisense).

### Metabolomics experiment

Intracellular metabolites were measured in MCF7 and HT29 cells treated with vitamin C using CE-TOFMS (Agilent Technologies, Palo Alto, CA, USA) as previously described^26^,^38^. In brief, the MCF7 and HT29 cells were seeded at a density of 4 × 10^5^ cells/well in 6-well plates. The cells were treated with vitamin C for 1 h, and then washed twice with 5% mannitol. Then, 600 μL of methanol containing the internal standards (25 μM each of methionine sulfone, ethane sulfonic acid, and D-Camphor-10-sulfonic acid) was added. The homogenate was mixed with 200 μL of Milli-Q water and 400 μL of chloroform. After centrifugation, the separated methanol-water layer was ultrafiltered using a Millipore 5-kDa cut-off filter to remove the proteins. The filtrate was lyophilized, dissolved in 25 μL of Milli-Q water and analyzed using CE-TOMS. The data obtained were analyzed using MasterHands^39^. The metabolite identities were determined by matching their *m/z* values and migration times with those of their standard compounds.

### Statistical analysis

The data were analyzed using the GraphPad Prism v 5.0 software (La Jolla, CA, USA). The statistical analysis of the experimental results was performed using the one-way analysis of variance (ANOVA). Data are presented as means ± standard deviation (SD) and differences with *P-*values < 0.05 were considered statistically significant.

## Additional Information

**How to cite this article**: Uetaki, M. *et al.* Metabolomic alterations in human cancer cells by vitamin C-induced oxidative stress. *Sci. Rep.* **5**, 13896; doi: 10.1038/srep13896 (2015).

## Supplementary Material

Supplementary Information
