# Supplementary material for: Metabolomic alterations in human cancer cells by vitamin C-induced oxidative stress
Source: Sci Rep. 2015 Sep 9;5:13896. doi: 10.1038/srep13896 (PMC4563566; doi:10.1038/srep13896)
Supplement: Supplementary Information [file srep13896-s1.pdf]

Metabolomic alterations in human cancer cells by vitamin C-induced oxidative stress

Megumi Uetaki[1, 2], Sho Tabata[1, 2*], Fumie Nakasuka[1, 3], Tomoyoshi Soga[1, 2, 3] and Masaru Tomita[1, 2, 3]

[1] *Institute for Advanced Biosciences, Keio University, 246-2 Mizukami, Kakuganji, Tsuruoka, Yamagata 997-0052, Japan*

[2] *Systems Biology Program, Graduate School of Media and Governance, Keio University, 5322 Endo, Fujisawa, Kanagawa 252-0882, Japan*

[3] *Environment and Information Studies, Keio University, 5322 Endo, Fujisawa, Kanagawa 252-0882, Japan*

*Correspondence and requests for materials should be addressed to S.T. (tabata@ttck.keio.ac.jp)

Supplementary Figure 1

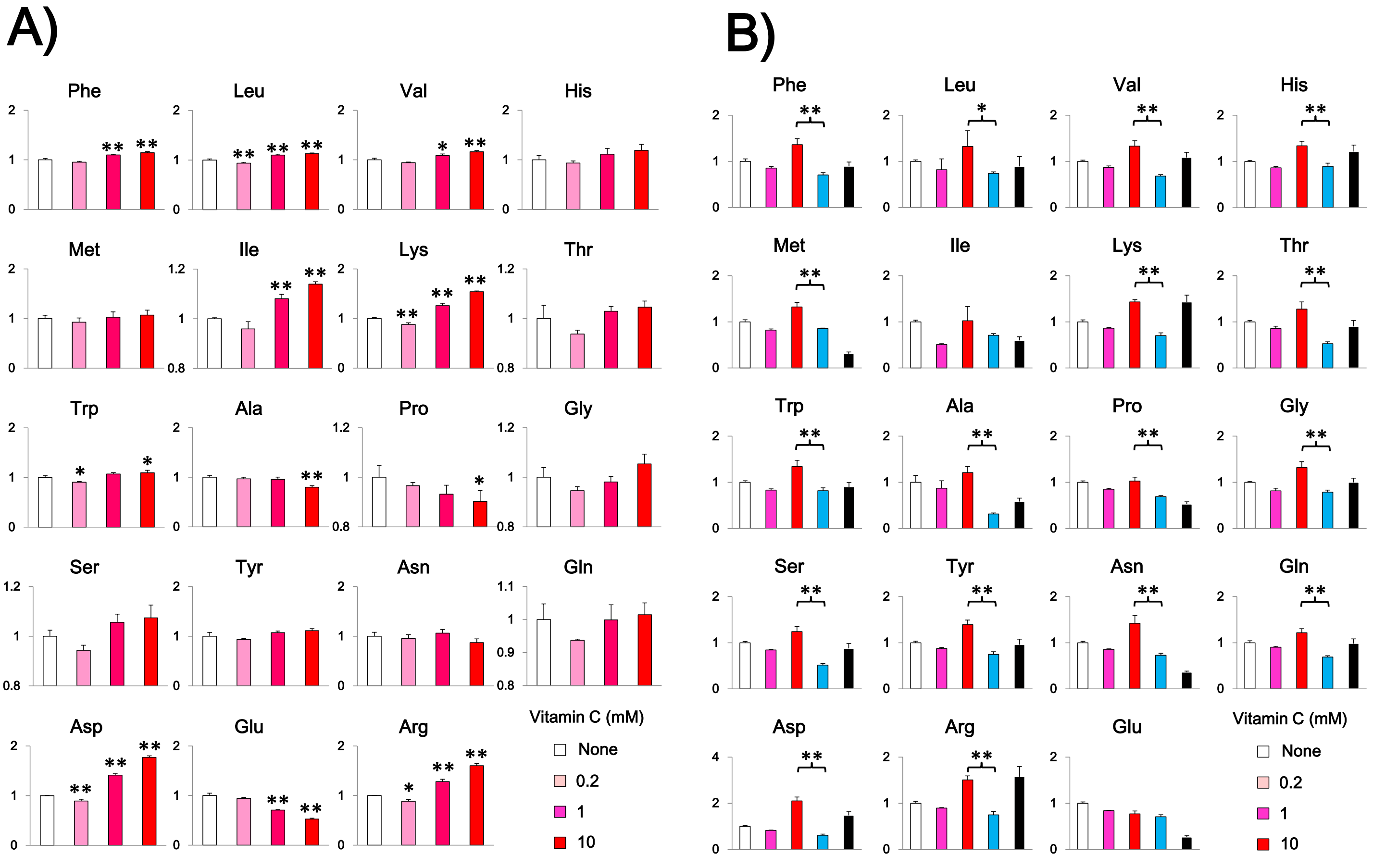

Supplementary Figure 1. Levels of amino acids in MCF7 cells treated with vitamin C.

A) Metabolic alterations in amino acid levels following treatment with vitamin C. MCF7 cells were incubated in DMEM without or with vitamin C, and metabolite levels were measured by CE-TOSMS. Bar graphs indicate the fold changes relative to the control sample (None).

B) Effects of NAC on the levels of amino acids in MCF7 cells stimulated with vitamin C. Bar graphs show the metabolite levels relative to the control (None).

Data are presented as means ± SDs from triplicate experiments. *$P < 0.05$, **$P < 0.01$.

Supplementary Figure 2

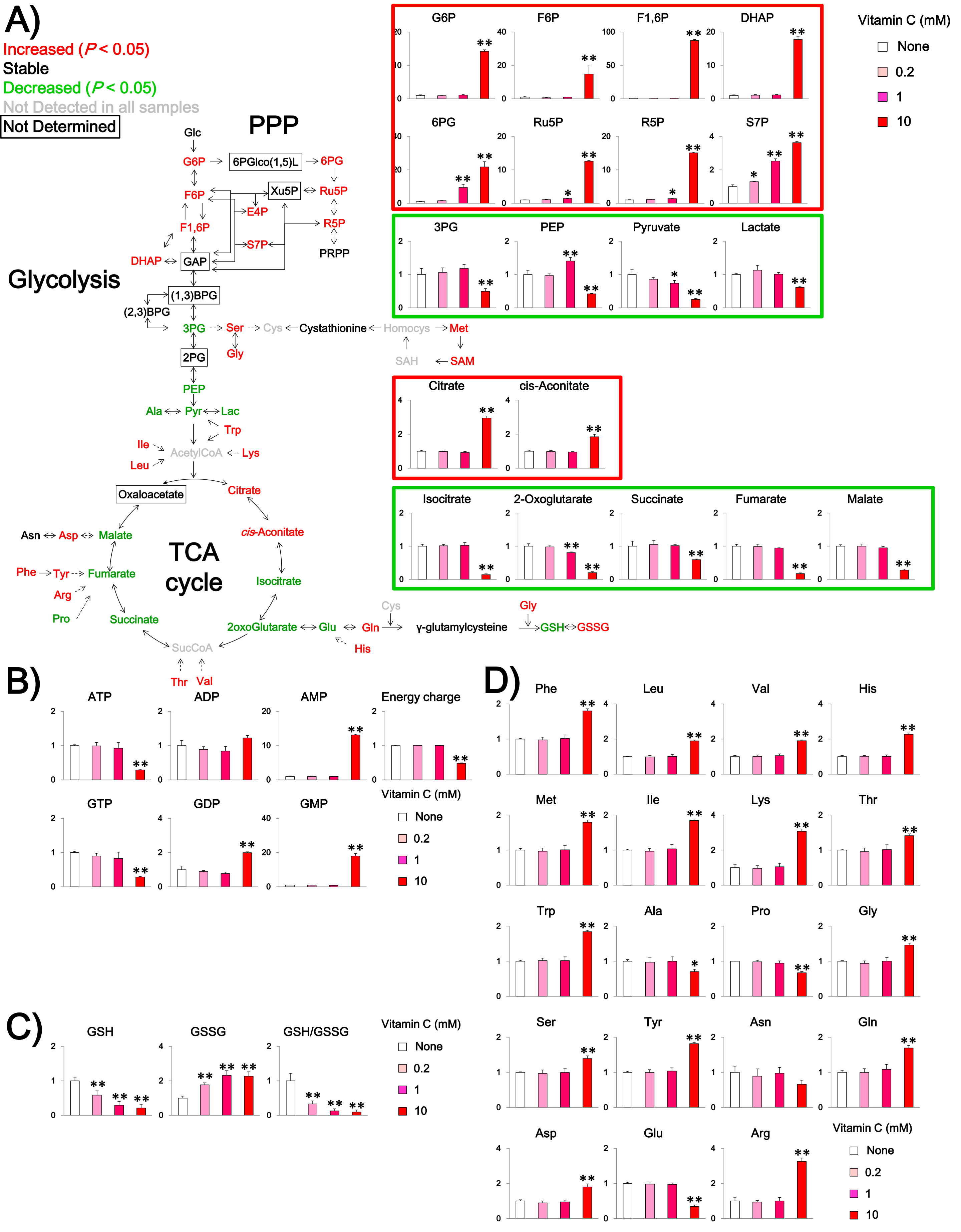

Supplementary Figure 2. Metabolic alterations induced by vitamin C in MCF7 cells.

A) Metabolic alterations in glycolysis and the TCA cycle induced by vitamin C. HT29 cells were incubated in DMEM without or with vitamin C, and metabolites levels were measured by CE-TOSMS. The colors of metabolites on the map indicate significant differences (red, upregulated; green, downregulated). Bar graphs indicate fold changes relative to the control sample (None). ND, not detected.

B) Effects of vitamin C on the levels of AMP, ADP, ATP, GMP, GDP, GTP, and energy charge. Bar graphs indicate the fold changes relative to the control sample (None). Energy charge: (ATP + 0.5 × ADP) / (ATP + ADP + AMP).

C) Effects of vitamin C on the levels of GSH and GSSG and the ratio of GSH to GSSG. Bar graphs show the metabolite levels relative to the control (None).

D) Metabolic alterations in amino acids by vitamin C. Bar graphs indicate the fold changes relative to the control sample (None).

Data are presented as means ± SDs from triplicate experiments. $^*P < 0.05$, $^{**}P < 0.01$.

Supplementary Figure 3

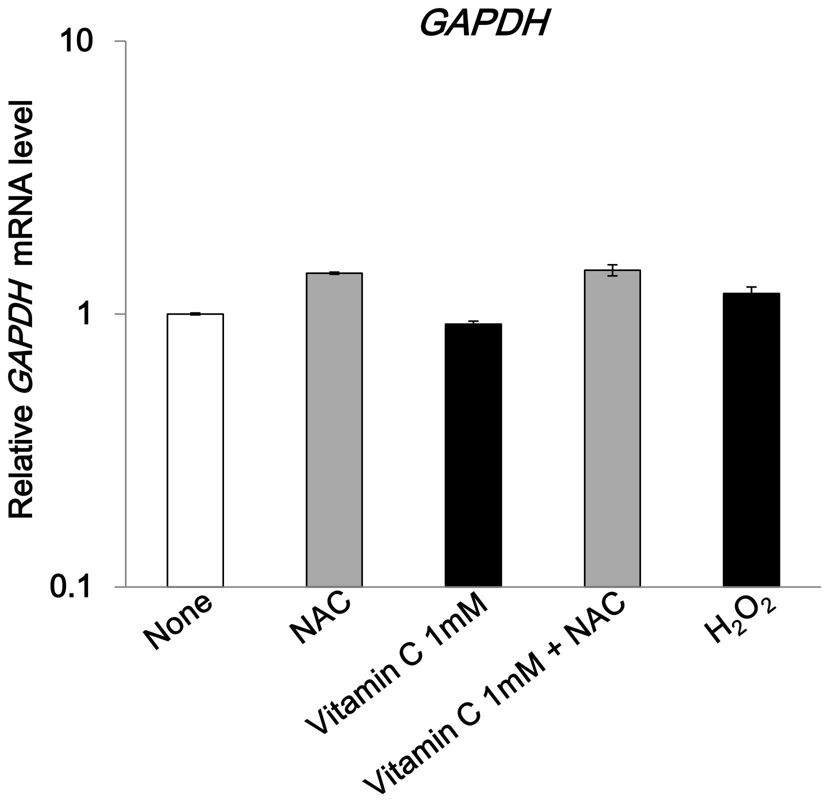

Supplementary Figure 3. Effects of vitamin C on *GAPDH* expression in MCF7 cells.
Cells were treated with vitamin C (1 mM), NAC (10 mM), and H_2O_2 (1 mM) for 24 h. Expression levels of *GAPDH* mRNA were measured using real-time PCR. Data are presented as means ± SDs from triplicate experiments.
